# Tamoxifen-Induced Cre-loxP Recombination Is Prolonged in Pancreatic Islets of Adult Mice

**DOI:** 10.1371/journal.pone.0033529

**Published:** 2012-03-28

**Authors:** Rachel B. Reinert, Jeannelle Kantz, Amanda Ackermann Misfeldt, Greg Poffenberger, Maureen Gannon, Marcela Brissova, Alvin C. Powers

**Affiliations:** 1 Department of Molecular Physiology and Biophysics, Vanderbilt University School of Medicine, Nashville, Tennessee, United States of America; 2 Division of Diabetes, Endocrinology and Metabolism, Department of Medicine, Vanderbilt University School of Medicine, Nashville, Tennessee, United States of America; 3 Department of Cell and Developmental Biology, Vanderbilt University School of Medicine, Nashville, Tennessee, United States of America; 4 Department of Veterans Affairs Tennessee Valley Healthcare System, Nashville, Tennessee, United States of America; University of Bremen, Germany

## Abstract

Tamoxifen (Tm)-inducible Cre recombinases are widely used to perform gene inactivation and lineage tracing studies in mice. Although the efficiency of inducible Cre-loxP recombination can be easily evaluated with reporter strains, the precise length of time that Tm induces nuclear translocation of CreER^Tm^ and subsequent recombination of a target allele is not well defined, and difficult to assess. To better understand the timeline of Tm activity in vivo, we developed a bioassay in which pancreatic islets with a Tm-inducible reporter (from *Pdx1^PB^*-*CreER^Tm^*;*R26R^lacZ^* mice) were transplanted beneath the renal capsule of adult mice previously treated with three doses of 1 mg Tm, 8 mg Tm, or corn oil vehicle. Surprisingly, recombination in islet grafts, as assessed by expression of the β-galactosidase (β-gal) reporter, was observed days or weeks after Tm treatment, in a dose-dependent manner. Substantial recombination occurred in islet grafts long after administration of 3×8 mg Tm: in grafts transplanted 48 hours after the last Tm injection, 77.9±0.4% of β-cells were β-gal+; in β-cells placed after 1 week, 46.2±5.0% were β-gal+; after 2 weeks, 26.3±7.0% were β-gal+; and after 4 weeks, 1.9±0.9% were β-gal+. Islet grafts from mice given 3×1 mg Tm showed lower, but notable, recombination 48 hours (4.9±1.7%) and 1 week (4.5±1.9%) after Tm administration. These results show that Tm doses commonly used to induce Cre-loxP recombination may continue to label significant numbers of cells for weeks after Tm treatment, possibly confounding the interpretation of time-sensitive studies using Tm-dependent models. Therefore, investigators developing experimental approaches using Tm-inducible systems should consider both maximal recombination efficiency and the length of time that Tm-induced Cre-loxP recombination occurs.

## Introduction

The advent of tamoxifen (Tm)-inducible Cre recombinases has greatly improved the ability to temporally control Cre-loxP recombination *in vivo*, and has been particularly useful for investigating properties of mature tissues in the adult mouse. Over the past decade, Tm-inducible gene recombination has been used to examine organ maintenance and function through a variety of approaches, including cell lineage tracing [1]–[12], inducible gene expression [13]–[17], and gene inactivation [18]–[21].

Several studies have described the importance of various parameters in Cre-loxP recombination, such as expression of Cre recombinase [22], [23] and the accessibility of loxP sites [24], [25]. For inducible Cre-loxP recombination, one critical parameter that is often poorly described is verification that the Tm dose used is appropriate for the experiment, not only for maximizing the spatial extent of recombination in the target tissue but also by limiting the temporal extent of recombination. Specifically, knowing the timeline of Tm-induced Cre-loxP recombination is critical for “pulse-chase” lineage tracing experiments, because a pulse that unknowingly extends into the chase period will continue to label newly generated cells, and lead to the interpretation that all labeled cells are derived from the cell population that existed during the expected pulse period. For example, the current limited knowledge of the Tm pulse period in adult mice may be one factor contributing to the discrepancies observed in recent lineage tracing studies of the pancreas.

In the pancreatic islet biology field, investigators are striving to understand the normal development and maintenance of insulin-producing β-cells and attempting to find sources for creating and regenerating β-cells, with the ultimate goal of treating diabetes. The use of Tm-inducible mouse models for cell lineage tracing has played a key role in advancing our understanding of pancreas biology, although some conflicting results have yet to be resolved. In an adaptation of classic pulse-chase experiments, Dor and colleagues were the first to use a Tm-inducible Cre driver strain to label mature pancreatic β-cells in adult mice [26]. After determining that the proportion of labeled β-cells did not change over time, they concluded that pre-existing β-cells were the source of β-cell replenishment in adult mice, and that there was no significant contribution of (unlabeled) stem cells to the β-cell population. This finding was also supported by studies using Tm-inducible systems to demonstrate that all mature β-cells possess a certain replication capacity [27]–[29]. In contrast, several other groups have used the same pulse-chase approach and concluded that β-cells can originate from non-β-cell sources, after observing changes in the proportion of labeled β-cells following post-injury regeneration or during pregnancy, when β-cell mass increases [30]–[32]. Further complicating our concept of β-cell turnover, one group reported isolating multipotent, stem-like cells from pancreatic islets, and, using the same lineage tracing methods as Dor et al., identified insulin+ cells as the source [33].

An alternative lineage tracing approach is to label a given cell type and determine whether or not it has the capacity to mature or transdifferentiate into another cell type. Using this more direct method of tracking cells, β-cells have been reported to originate from pancreatic ductal cells [34] and glucagon-producing α-cells [35] in the injured mature pancreas, suggesting that transdifferentiation may occur under specific circumstances. However, the mechanisms involved in this process still require further examination, as several studies have failed to find evidence of β-cell transdifferentiation from acinar cells [36]–[40] or ductal cells [41]–[44] in the adult pancreas.

While the Tm-inducible genetic models used in these studies have proven to be useful in addressing complex biological questions regarding pancreas development and maintenance, the conflicting results are sometimes difficult to reconcile. The fact that various Cre driver mice and reporter strains were used makes it inherently more difficult to compare different studies, as each model system differs in the specificity of Cre expression and the efficiency of target gene induction [45]–[47]. A recent report that described Tm-independent activity of Cre recombinase in the commonly used β-cell-targeted *RIP-CreER^Tm^* mouse found that the extent of the “leakiness” varied, depending on the target gene [48]. Moreover, a wide range of Tm doses and administration methods have been reported, even for studies using similar Cre driver and reporter mice. In fact, one review questioned whether different Tm doses could account for the conflicting outcomes in two very similar models of pancreatic ductal cell lineage tracing [46]. The need to draw such comparisons between experiments emphasizes the necessity for a full understanding of the parameters controlling recombination in each model system.

While understanding the temporal limits of CreER activity is crucial for the design and interpretation of lineage tracing experiments, the kinetics of Tm activity have been studied almost exclusively in embryonic tissues [49], [50]. Using immunohistochemistry, it was found that Cre localized to the nucleus of embryonic cells 24 hours after administration of Tm to a pregnant dam, but returned to the cytoplasm 48 hours after treatment [49]. Further studies showed that the extent of recombination events was dramatically different in embryos depending on the developmental stage at which Tm treatment was performed, due to the embryo's rapidly changing gene expression profile. These results suggested that Tm-induced recombination events in the embryo are restricted to a short time frame after drug administration to the dam [50]–[52]. Since most tissues in the adult animal do not show frequent, dramatic changes in gene expression or cell turnover, it is not possible to extrapolate the duration of active recombination following Tm administration in embryonic studies to that in adult mice.

In this study, we defined the kinetics of Tm-induced Cre-loxP recombination in pancreatic β-cells in adult mice. Because the precise level of circulating Tm necessary for inducing recombination *in vivo* is unknown, we used pancreatic islet transplantation as a bioassay to directly measure recombination at a given time following Tm administration. We found that significant recombination of reporter alleles can occur for weeks after Tm treatment, and that the length of time that a Tm pulse induces recombination is dose-dependent. Furthermore, we observed side effects in Tm-treated male mice that have not been reported in recent literature. These data have considerable implications for the design and interpretation of studies utilizing Tm-inducible systems in adult mice.

## Methods

### Ethics Statement

Animal studies were performed according to guidelines in the *Guide for the Care and Use of Laboratory Animals of the National Institutes of Health*. Animal protocols were approved by the Institutional Animal Care and Use Committee at Vanderbilt University Medical Center (Animal Welfare Assurance Number A3227-01). Surgeries were performed under ketamine/xylazine anesthesia, and all efforts were made to minimize pain and suffering.

### Mice

For the islet transplantation bioassay, male hemizygous transgenic *Pdx1^PB^*-*CreER^Tm^* [53] mice were bred with female *R26R^lacZ^* reporter mice (Jackson Laboratory, [54]) for one or two generations to obtain mice hemizygous for the Cre transgene and heterozygous or homozygous for the *R26R^lacZ^* reporter allele. For studies on Cre subcellular localization in the pancreas, we used *Pdx1^PB^*-*CreER^Tm^* mice that had been crossed with mice expressing a conditional (“floxed”) *Vegfa* allele, *Vegfa^loxP^* [55]. For initial experiments, we also used *RIP-CreER^Tm^* mice [26]. PCR genotyping was performed on tail biopsies with primers described [54]–[56]. A summary of the mouse strains used, along with the Mouse Genome Informatics (MGI) nomenclature, is shown in Table S1.

### Tamoxifen preparation and administration

Corn oil (Sigma C8267) was sterilized using a Steriflip vacuum-assisted filter unit (Millipore). Tamoxifen (Tm, Sigma T5648) was dissolved in filter-sterilized corn oil to make solutions of 10 mg/ml or 20 mg/ml, which were subsequently protected from light. Tm solutions were freshly prepared the day prior to each injection and placed on a nutator to dissolve overnight at room temperature. Before treatment, excess fur was shaved from the backs of recipient mice under isoflurane anesthesia. Recipient mice were then given subcutaneous injections of 1 mg Tm (100 µl volume), 8 mg Tm (400 µl volume), or corn oil vehicle every 48 hours, for a total of 3 doses over a 5-day period. Injection sites were sealed with Vetbond tissue adhesive (3 M) to prevent oil leakage. Following Tm or vehicle administration, mice were housed individually to prevent cross-contamination [57].

### Islet isolation and transplantation

Islet transplant experiments were performed with littermate donors and recipients, between 6 and 17 weeks of age. Islets were isolated from donor mice by collagenase P digestion [58] and handpicked to near 100% purity with microscopic guidance. Littermate recipient mice were anesthetized with a mixture of 90 mg/kg ketamine and 10 mg/kg xylazine (Henry Schein, Melville, NY) before islet transplantation was performed, as described previously [58]. Briefly, 50–150 freshly isolated islets from one donor mouse were transplanted under the kidney capsule of a single littermate recipient mouse either 48 hours, 1 week, 2 weeks, or 4 weeks after the final Tm or vehicle injection. Graft-bearing kidneys were harvested two weeks after islet transplantation.

### Tissue collection, immunohistochemistry, X-gal staining, and imaging

Islet graft-bearing kidneys and whole pancreata were dissected in ice-cold 10 mM PBS, fixed in 4% paraformaldehyde, equilibrated in 30% w/w sucrose, and frozen in Tissue-Tek Optimal Cutting Temperature compound (VWR Scientific Products, Willard, OH) before cryosectioning (detailed in [58]). Five-µm cryosections were labeled by immunohistochemistry as described previously [58], using the following primary antibodies: guinea pig anti-human insulin IgG (Linco Research, St. Charles, MO; diluted 1∶200), rabbit anti-β-galactosidase IgG (ICN Pharmaceuticals, Costa Mesa, CA; 1∶10000), and rabbit anti-Cre recombinase IgG (EMD4Biosciences, formerly Novagen; 1∶10000). Secondary antibodies conjugated to Cy2 or Cy3 fluorophores were obtained from Jackson ImmunoResearch Laboratories, Inc. (West Grove, PA). Slides with immunolabeled tissue were mounted with SlowFade Gold antifade reagent with DAPI nuclear counterstain (Invitrogen, Carlsbad, CA) before imaging. β-galactosidase activity was also assessed in tissue cryosections by X-gal staining, as described previously [58]. Tissue images were acquired using an Olympus BX41 fluorescence microscope (Olympus; Tokyo, Japan) and a Zeiss LSM 510 Meta laser scanning confocal microscope (Carl Zeiss; Jena, Germany).

Reproductive organs dissected from Tm- and vehicle-treated mice were fixed in 4% paraformaldehyde and dehydrated in an ethanol series (35%, 50%, and 70% for 30 minutes each) before embedding in paraffin. Five-µm sections were stained with hematoxylin and eosin. Tissue images were acquired with a ScanScope CS slide scanner (Aperio Technologies, Inc., Vista, CA).

### Quantification of recombined cells

Insulin+ and β-galactosidase+ (β-gal+) cells were counted manually with the aid of MetaMorph software (Universal Imaging, Downington, PA). At least three cross-sections were counted for each islet graft (200–3000 total insulin+ cells counted per graft), and at least ten islet cross-sections were counted per pancreas (300–500 insulin+ cells counted per mouse). The percentage of insulin+ β-cells expressing β-gal was calculated for each cross-section, and averaged for each graft or pancreas sample. Two to four tissue samples were obtained for each treatment group or time point.

## Results

### Tamoxifen-induced Cre-loxP recombination in pancreatic β-cells

Our interest in tamoxifen (Tm)-inducible systems began when developing a model in which we could inactivate production of vascular endothelial growth factor A (VEGF-A) by pancreatic islets in the adult mouse to evaluate its effects on islet vascularization and function. We initiated these experiments using two different mouse strains to target expression of a Tm-inducible Cre recombinase to pancreatic β-cells of adult mice, namely the *RIP*-*CreER^Tm^* [26] and *Pdx1^PB^*-*CreER^Tm^* [53] driver strains. As reported previously [48], we found that *RIP*-*CreER^Tm^*;*R26R^lacZ^* islets showed significant expression of the β-galactosidase reporter in the absence of Tm (Figure S1). Therefore, for subsequent experiments we used *Pdx1^PB^*-*CreER^Tm^* mice, crossed to the *R26R^lacZ^* reporter strain or to a strain containing a conditional *Vegfa* allele (*Vegfa^loxP^*, [55]).

### Tamoxifen-induced nuclear localization of Cre recombinase is time- and dose-dependent

To estimate the duration of Tm-induced Cre-mediated recombination in adult mice, we first evaluated nuclear localization of Cre recombinase in islet β-cells on pancreatic sections collected from transgenic *Pdx1^PB^*-*CreER^Tm^*;*Vegfa^loxP^* mice at different time points following the administration of 3×8 mg Tm. This dose effectively induced recombination of the floxed VEGF-A allele, as assessed by ELISA: islets isolated from *Pdx1^PB^*-*CreER^Tm^*;*Vegfa^loxP^* mice showed significantly reduced VEGF-A secretion one week and one month following Tm treatment (Figure S2).

**Figure 1:**
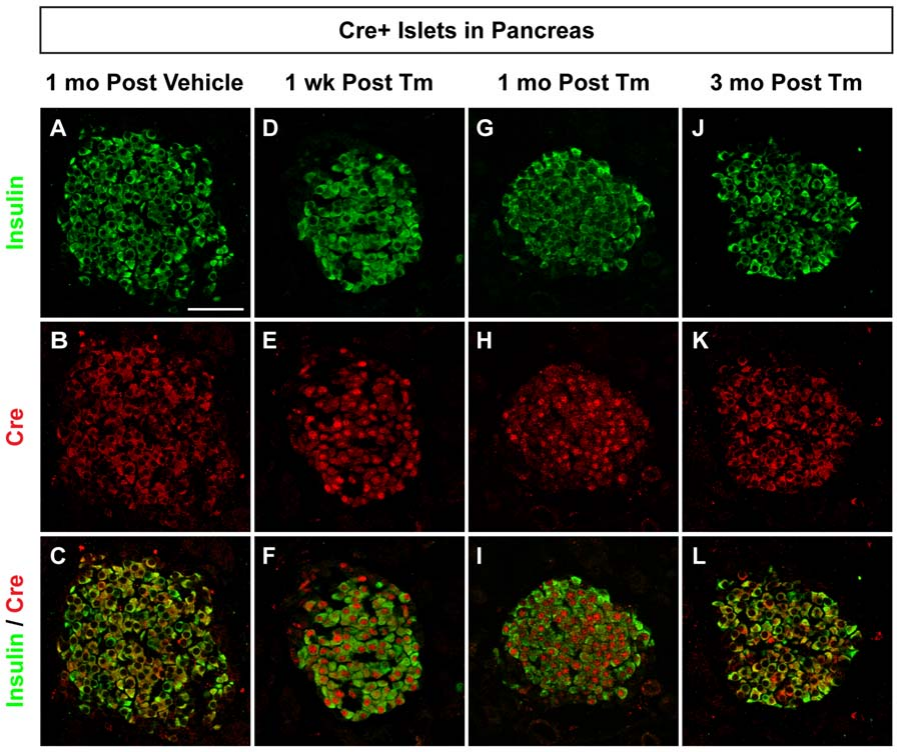
Tamoxifen-induced Cre subcellular localization is time-dependent. Representative islets from adult *Pdx1^PB^*-*CreER^Tm^*;*Vegfa^loxP^* mice given 8 mg tamoxifen (Tm, **D–L**) or corn oil vehicle (**A–C**) in three subcutaneous injections. Pancreata were harvested 1 week (**D–F**), 1 month (**G–I**), or 3 months (**J–L**) following the last injection and labeled with antibodies against insulin (green; **A**, **D**, **G**, **J**) and Cre recombinase (red; **B**, **E**, **H**, **K**). Merged images are shown in **C**, **F**, **I**, **L**. Scale bar in **A** is 50 µm, and applies to panels **B–L**.

Surprisingly, Cre was found in the β-cell nucleus and cytoplasm in *Pdx1^PB^*-*CreER^Tm^*;*Vegfa^loxP^* pancreas collected either one week or one month after the final Tm dose (Figure 1D–I). In contrast, vehicle-treated *Pdx1^PB^*-*CreER^Tm^*;*Vegfa^loxP^* mice displayed strict cytoplasmic localization of Cre in β-cells, as demonstrated by colocalization with insulin (Figure 1A–C). Likewise, β-cells from *Pdx1^PB^*-*CreER^Tm^*;*Vegfa^loxP^* mice sacrificed three months after the final Tm treatment demonstrated cytoplasmic but not nuclear Cre localization (Figure 1J–L). Extended nuclear localization of Cre was also observed five weeks after administration of 2×8 mg Tm to *Pdx1^PB^*-*CreER^Tm^*;*R26R^lacZ^* reporter mice, as demonstrated by colocalization of Cre and the nuclear marker DAPI in β-cells (Figure S3D–F). However, *Pdx1^PB^*-*CreER^Tm^*;*R26R^lacZ^* mice given 1×8 mg Tm showed cytoplasmic Cre localization at this time point (Figure S3A–C).

### Higher doses of tamoxifen induce recombination weeks following administration

**Figure 2:**
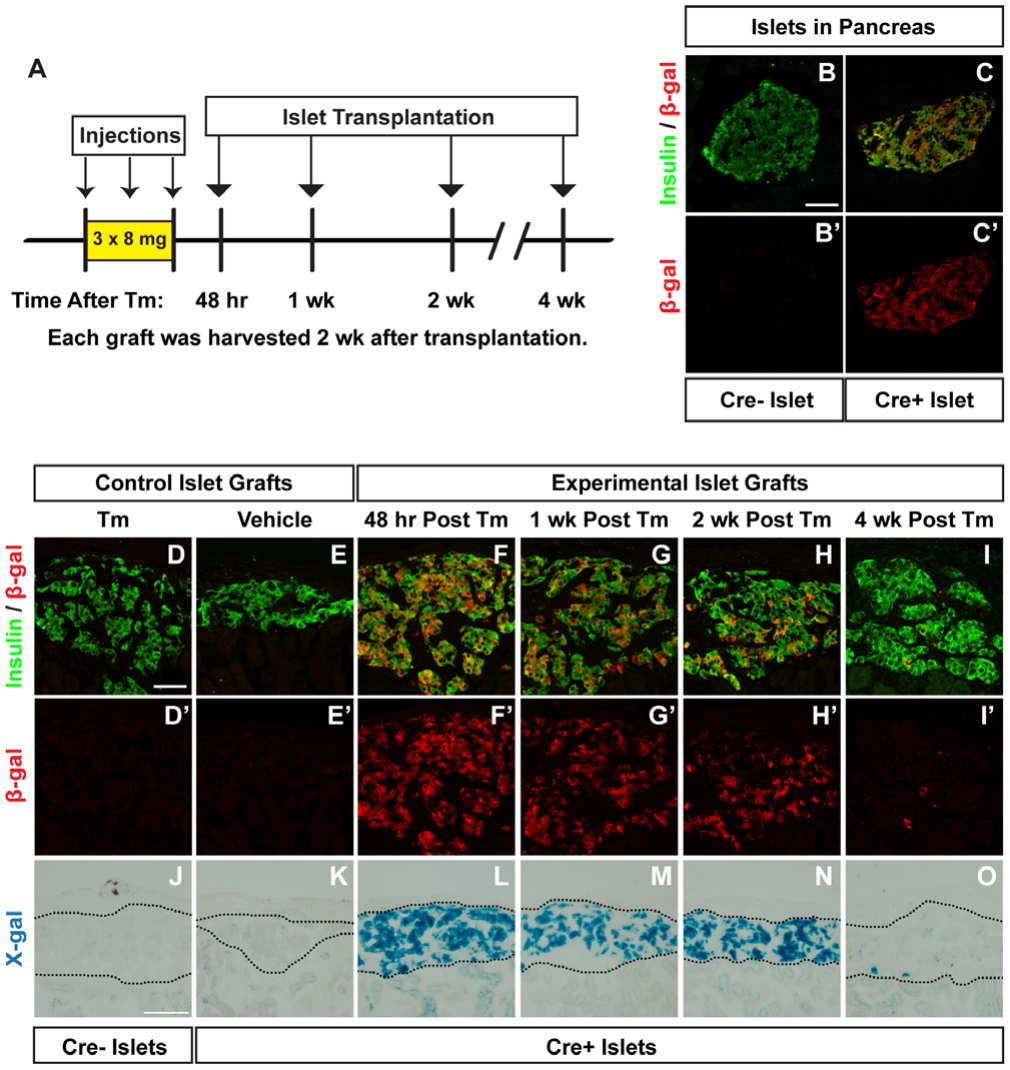
Higher dose tamoxifen induces recombination weeks following administration. **A.** Islets from untreated *Pdx1^PB^*-*CreER^Tm^*;*R26R^lacZ^* mice or *R26R^lacZ^* controls were transplanted into mice given three subcutaneous injections of 8 mg tamoxifen (Tm) or corn oil vehicle at the indicated times following the last injection. Pancreata and islet grafts were harvested 2 weeks after the final injection. **B–C.** Representative pancreatic islets from *R26R^lacZ^* (Cre−) mice (**B**) or *Pdx1^PB^*-*CreER^Tm^*;*R26R^lacZ^* (Cre+) mice (**C**) treated with 3×8 mg Tm. Cryosections were labeled with antibodies to insulin (green; **B**, **C**) and β-galactosidase (β-gal, red; **B**, **B′**, **C**, **C′**). Scale bar in **B** is 50 µm, and applies to panels **B′**, **C**, **and C′**. **D–I**. Islet graft cryosections were labeled with antibodies to insulin (green; **D–I**) and β-gal (red; **D–I**, **D′–I′**). Scale bar in **D** is 50 µm, and applies to panels **E–I**, **D′–I′**. **J–O**. β-gal activity was tested in islet grafts using X-gal. Scale bar in **J** is 100 µm, and applies to panels **K–O**. Images of the full graft cross-sections (before cropping and rotating for visual clarity) are shown in Figures S4 and S5.

**Figure 3:**
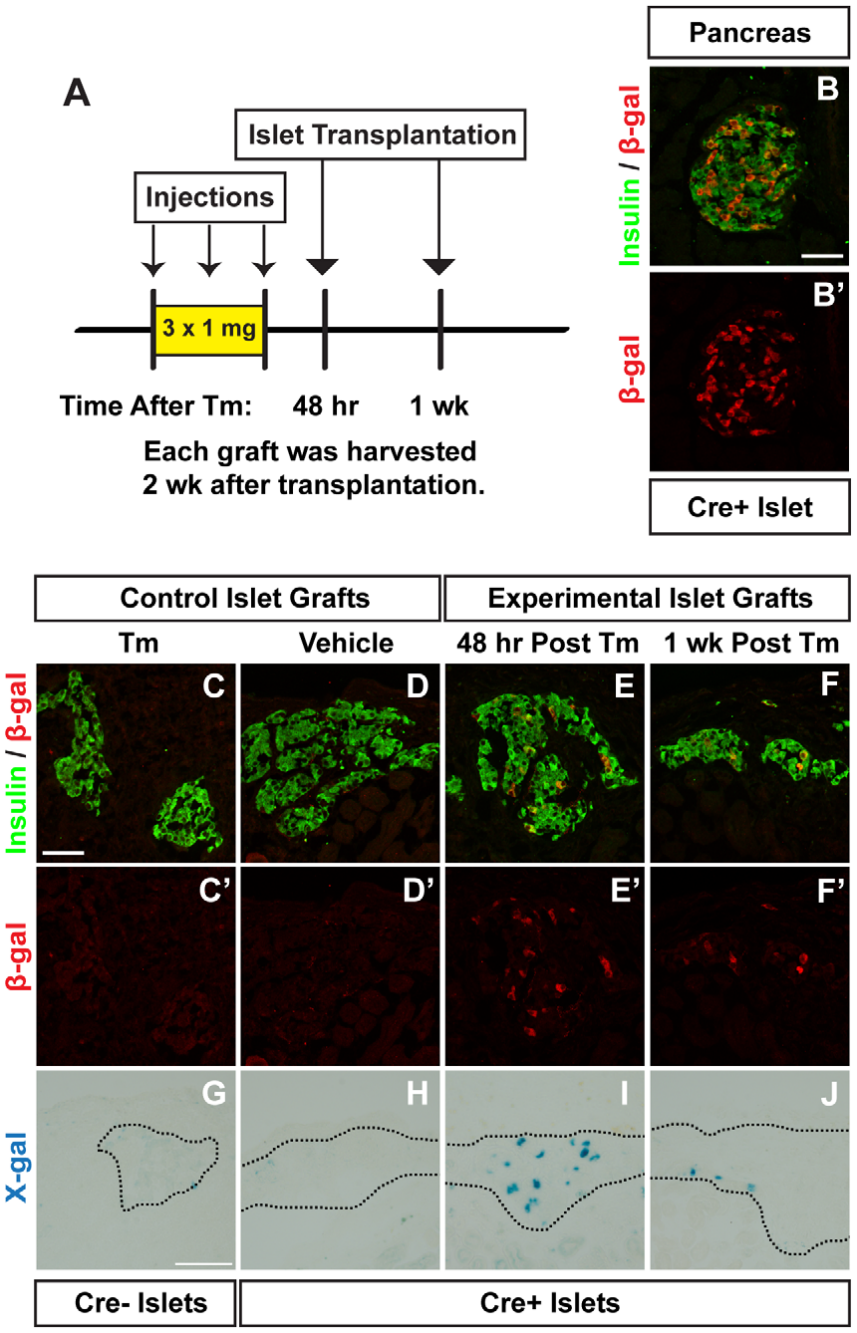
Lower dose tamoxifen induces recombination up to one week following administration. **A.** Islets from untreated *Pdx1^PB^*-*CreER^Tm^*;*R26R^lacZ^* (Cre+) mice or *R26R^lacZ^* (Cre−) controls were transplanted into mice given three subcutaneous injections of 1 mg tamoxifen (Tm) or corn oil vehicle at the indicated times following the last injection. Pancreata and islet grafts were harvested 2 weeks after the final injection. **B.** Representative islet from a Cre+ mouse treated with 3×1 mg Tm, labeled with antibodies to insulin (green; **B**) and β-galactosidase (β-gal, red; **B**, **B′**). Scale bar in **B** is 50 µm, and applies to panel **B′**. **C–F**. Islet graft cryosections were labeled with antibodies to insulin (green; **C–F**) and β-gal (red; **C-F**, **C′–F′**). Scale bar in **C** is 50 µm, and applies to panels **D–F**, **C′–F′**. **G–J**. β-gal activity was tested in islet grafts using X-gal. Scale bar in **G** is 100 µm, and applies to panels **H–J**. Images of the full graft cross-sections (before cropping and rotating for visual clarity) are shown in Figures S6 and S7.

To better understand how long a given Tm dose is able to induce Cre-mediated recombination *in vivo*, we developed a system that would allow us to evaluate recombination that occurs at any given time following Tm treatment. We reasoned that measuring the serum Tm concentration in this model would likely be unhelpful, because the precise level of circulating Tm necessary for inducing recombination is unknown. Therefore, we designed a bioassay using pancreatic islet transplantation to assess recombination in Tm-naïve islet β-cells transplanted into Tm-treated mice. In this model, islets containing a Tm-inducible Cre reporter (from *Pdx1^PB^*-*CreER^Tm^*;*R26R^lacZ^* mice) were transplanted beneath the renal capsule of mice treated with different doses of Tm prior to islet transplantation (Figures 2A and 3A). In this way, the relevant endpoint, recombination, served as the metric.

We first evaluated a dose of 3×8 mg Tm, a dose similar to that used in other studies for lineage tracing of β-cells in adult mice [26]–[28], [30]–[33], [35]. As expected, islets in Tm-treated *R26R^lacZ^* mice did not express β-gal, as visualized by immunohistochemistry (Figure 2B). In *Pdx1^PB^*-*CreER^Tm^*;*R26R^lacZ^* mice given 3×8 mg Tm, 80.1±8.2% of β-cells in pancreatic islets expressed β-gal (Figure 2C). To evaluate this dose in the transplant model, recipient mice were given three subcutaneous injections of Tm or vehicle before receiving a pancreatic islet transplant from Tm-naïve *Pdx1^PB^*-*CreER^Tm^*;*R26R^lacZ^* donor mice. Islet grafts were placed 48 hours, 1 week, 2 weeks, or 4 weeks following the final Tm injection (Figure 2A). We observed significant recombination in *Pdx1^PB^*-*CreER^Tm^*;*R26R^lacZ^* islet grafts in mice given 3×8 mg Tm for weeks after the final Tm dose. When quantified, 77.9±0.4% of β-cells expressed β-gal when transplanted 48 hours after the final Tm injection (Figure 2F, Figure S4E–F), 46.2±5.0% were β-gal+ 1 week after injection (Figure 2G, Figure S4G–H), and 26.3±7.0% expressed β-gal 2 weeks after injection (Figure 2H, Figure S4I–J). Recombination was also noted in grafts placed 4 weeks after the final Tm injection, with 1.9±0.9% of β-cells positive for β-gal (Figure 2I, Figure S4K–L). In contrast, islet cells from *R26R^lacZ^* mice transplanted into Tm-treated mice did not express β-gal (Figure 2D, Figure S4A–B). Similarly, most *Pdx1^PB^*-*CreER^Tm^*;*R26R^lacZ^* islet cells transplanted into vehicle-treated mice did not show signs of recombination (Figure 2E, Figure S4C–D), although a few β-gal+ β-cells were found in one of the grafts (0.6±0.6%). These results were confirmed by X-gal staining (Figure 2J–O, Figure S5).

### Lower doses of tamoxifen induce recombination up to one week following administration

Next, we used the islet transplantation bioassay to test the duration of recombination following 3×1 mg Tm (Figure 3A), a dose that also induced effective recombination in *Pdx1^PB^*-*CreER^Tm^*; *Vegfa^loxP^* mice (manuscript in preparation). Compared to mice receiving 3×8 mg Tm, treatment with 3×1 mg Tm induced less recombination in pancreatic islets of *Pdx1^PB^*-*CreER^Tm^*;*R26R^lacZ^* mice (29.8±4.1% of β-cells expressed β-gal, Figure 3B). Likewise, we observed less, but notable, recombination in *Pdx1^PB^*-*CreER^Tm^*;*R26R^lacZ^* grafts placed in mice receiving 3×1 mg Tm: 4.9±1.7% of β-cells expressed β-gal 48 hours after Tm (Figure 3E, Figure S6E–F), and 4.5±1.9% of β-cells were β-gal+ 1 week after Tm (Figure 3F, Figure S6G–H). No β-gal+ β-cells were noted in control grafts (Figure 3C–D, Figure S6A–D). These results were also confirmed by X-gal staining of the islet grafts (Figure 3G–J, Figure S7).

### Duration of tamoxifen-induced gene recombination is dose-dependent

**Figure 4:**
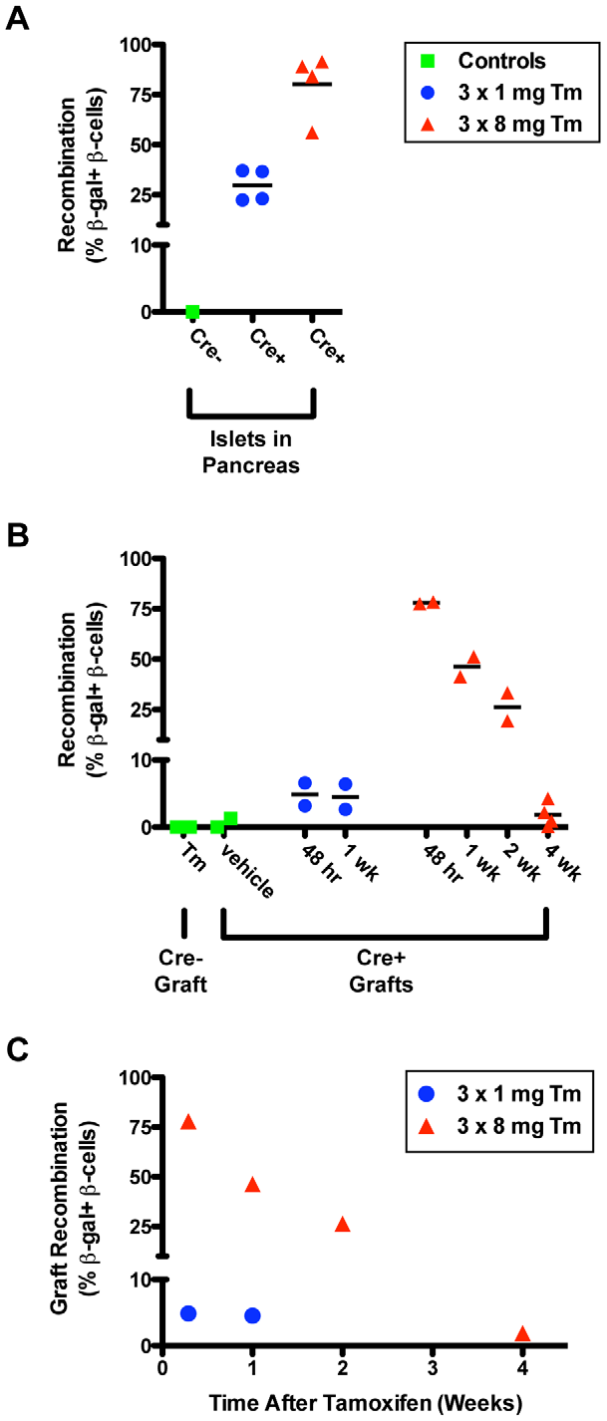
Duration of tamoxifen-induced gene recombination is dose-dependent. The percentage of insulin+ β-cells expressing β-gal in islets from pancreas sections (**A**) and from transplanted islet grafts (**B**) is shown. Each data point represents the percentage of double-positive cells to all insulin+ β-cells counted in a single mouse sample. Cre−, *R26R^lacZ^* control islets; Cre+, *Pdx1^PB^*-*CreER^Tm^*;*R26R^lacZ^* islets; Tm, tamoxifen. **C**. Amount of recombination observed in islet grafts from mice given either 3×1 mg or 3×8 mg tamoxifen at the indicated time points following the last tamoxifen injection. Data in **C** is expressed as means of data in **B**.

A summary of Tm-induced recombination in *Pdx1^PB^*-*CreER^Tm^*;*R26R^lacZ^* pancreatic islets and islet grafts is shown in Figure 4. Recombination of β-cells in both endogenous pancreatic islets (Figure 4A) and in islet grafts (Figure 4B) is dose-dependent. At either dose, recombination in the transplanted islet cells was lower than recombination in pancreatic islets. However, all groups of *Pdx1^PB^*-*CreER^Tm^*;*R26R^lacZ^* islets transplanted into Tm-treated mice showed recombination rates higher than vehicle-treated controls, independent of Tm dose or time of transplantation. While recombination in *Pdx1^PB^*-*CreER^Tm^*;*R26R^lacZ^* islet grafts from mice receiving 3×1 mg Tm was relatively low at both the 48-hour and 1-week post-treatment time points, compared to islets in the pancreas of mice receiving 3×1 mg Tm, the recombination seen in *Pdx1^PB^*-*CreER^Tm^*;*R26R^lacZ^* islet grafts from mice receiving 3×8 mg Tm was substantial for 2 weeks following Tm administration (Figure 4B). Importantly, *Pdx1^PB^*-*CreER^Tm^*;*R26R^lacZ^* islet grafts from mice treated with 3×8 mg Tm showed recombination at a rate higher than controls even 4 weeks after treatment (Figure 4B). The estimated kinetics of Tm-induced recombination in our model system are plotted in Figure 4C.

### Long-term side effects of tamoxifen treatment

At both doses, we observed major side effects in Tm-treated mice that have not been mentioned in recent publications. The corn oil vehicle itself was incompletely absorbed in some mice, as indicated by subcutaneous masses found in the area of injection in both vehicle-treated and Tm-treated mice (Figure S8A). Upon dissection, these masses contained pockets of transparent oil, with no signs of inflammation or infection. Oil pockets were especially prevalent in female mice, and lasted for weeks or months after Tm administration. Additionally, male mice treated with Tm, but not corn oil vehicle alone, experienced dramatic scrotal enlargement (Figure S8B). This enlargement was first observed one week after Tm administration and lasted for at least three months (our last observed time point). Almost all of the >100 male mice we have treated with Tm developed this abnormality to some degree. While higher Tm doses did cause more dramatic anatomic changes, variability was seen between mice given identical doses. Some mice were more bothered by the scrotal changes, and developed self-inflicted wounds. Upon dissection, no changes were observed in the size of male reproductive organs or perigonadal fat pads. However, the fat appeared to envelop the testes more loosely in Tm-treated mice, and in some cases, loops of intestine were found to extend into the scrotum, indicating herniation. Histological examination of the male reproductive tract revealed no changes in reproductive organs, such as testis and epididymis, following Tm treatment (Figure S8C–D). Instead, Tm-treated males showed patches of edematous reactive fat containing spindle cells (Figure S8F). One mouse with extensive self-inflicted wounds showed chronic inflammation and fat necrosis within the preputial gland, located in the subcutaneous tissue adjacent to the penis (Figure S8H).

## Discussion

Tm-inducible Cre-loxP systems are being used in broad areas of research and are providing important biologic insights in tissue development, maintenance, and function. However, our understanding of the parameters involved in recombination is incomplete. One unresolved issue regarding Cre-loxP recombination in adult mice is the length of time that Tm induces recombination. In this study, we developed an *in vivo* bioassay using pancreatic islet transplantation to directly measure recombination at specific times following Tm administration, thereby quantifying the biologic and pharmacologic half-life of Tm. We found that: (1) administration of high Tm doses leads to extended CreER nuclear localization; (2) Tm administration induces reporter gene recombination for several days or weeks after Tm treatment is completed, depending on the original dose administered; and (3) Tm treatment induces side effects that may have physiologic consequences in Tm-inducible models.

Multiple factors are involved in obtaining specific and effective Cre-loxP recombination *in vivo*, including (but not limited to) the promoter or enhancer driving expression of Cre recombinase, the accessibility of loxP sites in the target gene, and, for inducible systems, the Tm dose used [23], [48], [49], [59]. First, the chosen promoter or enhancer in a Cre driver mouse ideally targets Cre expression to specific cell types; in reality, transgenic mice do not express Cre in 100% of the targeted cells, and some transgenes show aberrant expression of Cre in untargeted cell types [60]. Second, the accessibility of target loxP alleles to Cre recombinase can also impact recombination efficiency. For example, recombination of the *Z/AP* and *Z/EG* reporters is less efficient than recombination of reporter alleles in the *ROSA26* locus [25], [61], and studies with these reporters may not necessarily reflect recombination of other target alleles [48]. Third, in addition to the wide range of Tm doses reported in the literature, there are a variety of Tm administration methods, from the use of Tm or its active metabolite 4-hydroxytamoxifen, to the drug preparation (oil suspensions vs. implanted pellets), and the route of administration (subcutaneous vs. intraperitoneal vs. oral). Finally, our bioassay data demonstrates that the duration of Tm activity is another important variable to consider for Tm-inducible systems. In all, this wide range in model systems and methodology makes it difficult to compare different studies, and may contribute to the conflicting results reported in the pancreatic β-cell literature.

We developed this bioassay to directly assess the length of time that a given dose of Tm can induce Cre-loxP recombination *in vivo*. Early studies on Tm-induced Cre-loxP recombination utilized the rapid turnover of keratinocytes in epidermis to show that induction of reporter gene expression was limited to a few days after administration of relatively low doses of Tm [22], [62]. Reporter gene-expressing keratinocytes that originated in the basal epidermal layer were found to have migrated out within one week of stopping Tm treatment, leaving unlabeled, newly generated cells below. Additionally, subcellular localization of Cre recombinase was correlated with Tm administration, as Cre was found in the keratinocyte nucleus in mice currently undergoing Tm treatment, but was localized to the cytoplasm three days after the final Tm dose.

In contrast to the doses used in those early experiments to evaluate the timeline of Tm activity in skin (which were similar to the low dose used in our bioassay) [22], [62], many recent studies have used much higher Tm doses to achieve maximal recombination of reporter alleles in target tissues [6]–[10], [12]. The rationale for using a higher Tm dose in studies utilizing Tm-inducible models in the pancreas [26]–[28], [30]–[33], [35] include: (1) the transgenic mice commonly used to target pancreatic β-cells (*RIP*-*CreER^Tm^* [26] and *Pdx1^PB^*-*CreER^Tm^* [53]) were generated using the *CreER^Tm^* sequence [51], which has been shown to be less sensitive to Tm than the *CreER^T2^* sequence [63]; and (2) recombination efficiency is influenced by the accessibility of target loxP alleles to Cre recombinase [48]. The *Z/AP* reporter mouse has been frequently used to label pancreatic β-cells, and recombination of the *Z/AP* and *Z/EG* reporter alleles is less efficient than recombination of reporter alleles in the *ROSA26* locus [25]. Although using the Tm dose that allows for maximal recombination is desirable, our data show that high doses of Tm can induce a prolonged period of Tm-induced Cre activity and unwanted side effects. Therefore, determining the optimal Tm dose for efficacy and safety will be particularly important in characterizing new model systems, including the recently described *MIP-CreER* mouse that targets β-cells [60].

We examined the subcellular localization of Cre recombinase to estimate the time frame of potential recombination following Tm treatment. The fact that Cre was detected in nuclei of β-cells in the pancreas up to five weeks following the final Tm dose suggested that administration of Tm to adult mice induces a period of recombination that is much longer than the 12- to 48-hour window originally described in Tm-treated embryos [49], [50], [52]. Indeed, we observed significant Tm-induced recombination up to four weeks following drug administration, though perhaps to a lower extent than the number of β-cells showing nuclear localization of Cre might suggest. There are several possible reasons for this discrepancy in Cre subcellular localization and target gene recombination. We analyzed Cre localization in endogenous β-cells in the pancreas of transgenic mice, while the islet grafts had been transplanted under the kidney capsule of recipient mice. There may be differences in Tm availability at these two sites, as the pancreas was found to have unexpectedly high concentrations of Tm compared to other tissues [64], [65]. Additionally, the low blood flow experienced by newly transplanted islets may reduce the amount of Tm circulating to the β-cells, as revascularization of islet grafts takes several days to weeks to occur [58]. For these reasons, our islet graft model may in fact underestimate the amount of recombination that may occur in endogenous β-cells of Tm-treated mice. Alternatively, there may not be a direct correlation between Cre localization and recombination of target alleles; however, this is difficult to assess, as the amount of nuclear Cre required for inducing recombination is dependent on the sensitivity of the targeted floxed allele [25], [48], and may not necessarily be detected by immunohistochemistry. Importantly, our data shows that evaluating Cre subcellular localization alone is not sufficient to estimate active recombination in a given cell.

We administered Tm subcutaneously, as other groups have reported without noting side effects [28], [44], [66]. While unexpected, the oil pockets we observed are consistent with a report that described incomplete absorption of oil vehicles after an attempt to administer hormones subcutaneously to rodents [67]. In that study, the investigators collected the subcutaneous oil that remained days or weeks after injection and found that it still contained biologically active estrins. This observation raises the possibility that Tm itself is slowly and/or incompletely absorbed following subcutaneous injection, which could lead to the prolonged biologic activity we observed in this study, and potentially to variability in Tm dosing between mice. Alternative Tm administration methods, such as implanted or food-based pellets, will prevent side effects associated with the oil vehicle. However, the length of Tm action must still be determined for each of these treatment protocols.

The side effects that we observed in Tm-treated male mice were also surprising, because there has been little discussion of adverse events following Tm administration to adult transgenic mice [24], [68]–[70]. The pathologic changes in the scrota of Tm-treated mice appear to be limited to fat (and in one case, the preputial gland), and not to male reproductive organs. Instead, the scrotal swelling in Tm-treated mice closely resembles the scrotal hernias observed in male mice treated with estrogenic compounds, which were associated with hypertrophy of inguinal and scrotal skeletal muscle [71]–[73]. As Tm has mainly estrogenic actions in mice [64], the hernias may be an indication that Tm treatment is promoting feminization of male mice. This is potentially concerning for investigators using these models for studies on metabolic diseases like diabetes. It is well known that wild-type male and female mice show differences in glucose tolerance [74], [75], and some recent studies have reported that male mice are more susceptible to developing glucose intolerance following Cre-loxP-mediated inactivation of critical β-cell genes such as FoxM1 and Ngn3 [76], [77]. Thus, it is important not only to include Tm-treated control groups to evaluate the drug's physiologic effects on the mice, but also to limit the Tm dose in order to minimize these effects.

In summary, we observed a prolonged period of Tm-induced nuclear localization of Cre recombinase, accompanied by significant levels of Cre-loxP recombination days and weeks after Tm treatment. These findings have important implications for the design and interpretation of experiments utilizing Tm-inducible systems. While a prolonged period of recombination is not necessarily undesirable in studies in which Tm is used to inactivate target genes, it is a critical parameter in lineage tracing experiments that rely on the induction of a reporter gene during a defined “pulse” period. Because many variables may affect Tm-induced recombination in a given model, the doses and timeline we describe cannot be applied as strict guidelines for all Tm-inducible systems. Importantly, these data caution against the use of high Tm doses with the expectation that the effects of Tm are innocuous and short-lived. We recommend that investigators carefully define the Tm dose and duration of action in each model system.

## Supporting Information

Figure S1 **Tamoxifen-independent recombination in** ***RIP*** **-** ***CreER^Tm^*** **;** ***R26R^lacZ^*** **mice.** Pancreas was harvested from adult *RIP*-*CreER^Tm^*;*R26R^lacZ^* mice not exposed to tamoxifen (Tm). Immunofluorescence was performed on cryosections for insulin (blue; **A**, **C**) and β-galactosidase (β-gal, red; **B**, **C**). Scale bar in **A** is 50 µm, and applies to panels **B–C**.
(TIF)

Figure S2 **VEGF-A expression is significantly reduced in** ***Pdx1^PB^*** **-** ***CreER^Tm^*** **;** ***Vegfa^loxP^*** **islets after administration of 3×8 mg tamoxifen.** Islets were isolated from adult *Pdx1^PB^*-*CreER^Tm^*;*Vegfa^loxP^* mice and *Vegfa^loxP^* controls before tamoxifen (Tm) treatment and 1 week and 1 month following 3 doses of 8 mg Tm. Aliquots of 70 size-matched islets were cultured in 500 µl RPMI-1640 media for 48 hours at 37°C, and VEGF-A in the cultured media was measured by ELISA (R&D Systems) as described [78]. **P<0.0001.
(TIF)

Figure S3 **Tamoxifen-induced Cre subcellular localization is dose-dependent.** Representative islets from adult *Pdx1^PB^*-*CreER^Tm^*;*R26R^lacZ^* mice given one (**A–C**) or two (**D–F**) subcutaneous injections of 8 mg tamoxifen (Tm). Pancreata were harvested 5 weeks following the last injection and labeled with antibodies against insulin (green; **C**, **F**) and Cre recombinase (red; **B**, **C**, **E**, **F**); DAPI nuclear stain (blue; **A**, **C**, **D**, **F**). Scale bar in **A** is 50 µm, and applies to panels **B–F**.
(TIF)

Figure S4 **Higher dose tamoxifen induces recombination weeks following administration.** Images of the full graft cross-sections shown in Figure 2, before cropping and rotating for visual clarity. Scale bar in **A** is 200 µm, and applies to panels **B–L**.
(TIF)

Figure S5 **Higher dose tamoxifen induces recombination weeks following administration.** Islets from untreated *Pdx1^PB^*-*CreER^Tm^*;*R26R^lacZ^* mice (Cre+) or *R26R^lacZ^* controls (Cre−) were transplanted into mice given three subcutaneous injections of 8 mg tamoxifen (Tm) or corn oil vehicle at the indicated times following the last injection. Islet grafts were harvested 2 weeks after transplantation and subjected to X-gal staining (**A, C, E, G, I, K, M**). Phase contrast images with color overlay are shown in **B**, **D**, **F**, **H**, **J**, **L**, and **N**. Scale bar in **A** is 200 µm, and applies to panels **B–N**. Panels **C**, **E**, **G**, **I**, **K**, and **M** are images of the full graft cross-sections shown in Figure 2, before cropping and rotating for visual clarity.
(TIF)

Figure S6 **Lower dose tamoxifen induces recombination up to one week following administration.** Images of the full graft cross-sections shown in Figure 3, before cropping and rotating for visual clarity. Scale bar in **A** is 200 µm, and applies to panels **B–H**.
(TIF)

Figure S7 **Lower dose tamoxifen induces recombination up to one week following administration.** Islets from untreated *Pdx1^PB^*-*CreER^Tm^*;*R26R^lacZ^* mice (Cre+) or *R26R^lacZ^* controls (Cre−) were transplanted into mice given three subcutaneous injections of 1 mg tamoxifen (Tm) or corn oil vehicle at the indicated times following the last injection. Islet grafts were harvested 2 weeks after transplantation and subjected to X-gal staining (**A, C, E, G, I, K, M, O**). Phase contrast images with color overlay are shown in **B**, **D**, **F**, **H**, **J**, **L**, **N**, and **P**. Scale bar in **A** is 200 µm, and applies to panels **B–P**. Panels **E**, **G**, **I**, **K**, **M**, **and O** are images of the full graft cross-sections shown in Figure 3, before cropping and rotating for visual clarity.
(TIF)

Figure S8 **Side effects of tamoxifen treatment.** **A.** Some mice given subcutaneous injections of corn oil vehicle with or without tamoxifen (Tm) had subcutaneous accumulation of oil (white arrows) weeks and months following the last oil injection. **B.** Tm-treated male mice demonstrated scrotal enlargement (black arrows) as early as one week after treatment and lasting for months. Tm-treated mice shown were given 3×8 mg Tm, but similar results were observed in mice given 3×1 mg Tm. **C–H.** H&E-stained sections of tissue from the scrota of control (**C, E, G**) and Tm-treated (**D, F, H**) male mice. Images were acquired with a ScanScope CS slide scanner. **C–D**. Seminiferous tubules of the testes. Scale bar in **D** is 200 µm, and applies to panel **C**. **E–F**. Scrotal fat in a control mouse (**E**), compared to the reactive fat observed in a Tm-treated mouse (**F**). Scale bar in **F** is 200 µm, and applies to panel **E**. **G–H**. One Tm-treated mouse showed chronic inflammation and fat necrosis within the preputial gland (**H**); control gland, **G**. Scale bar in **H** is 400 µm, and applies to panel **G**.
(TIF)

Table S1 **Summary of mouse strains.**
(DOC)
