# Supplementary figures and images for: Tamoxifen-Induced Cre-loxP Recombination Is Prolonged in Pancreatic Islets of Adult Mice
Source: PLoS One. 2012 Mar 28;7(3):e33529. doi: 10.1371/journal.pone.0033529 (PMC3314663; doi:10.1371/journal.pone.0033529)

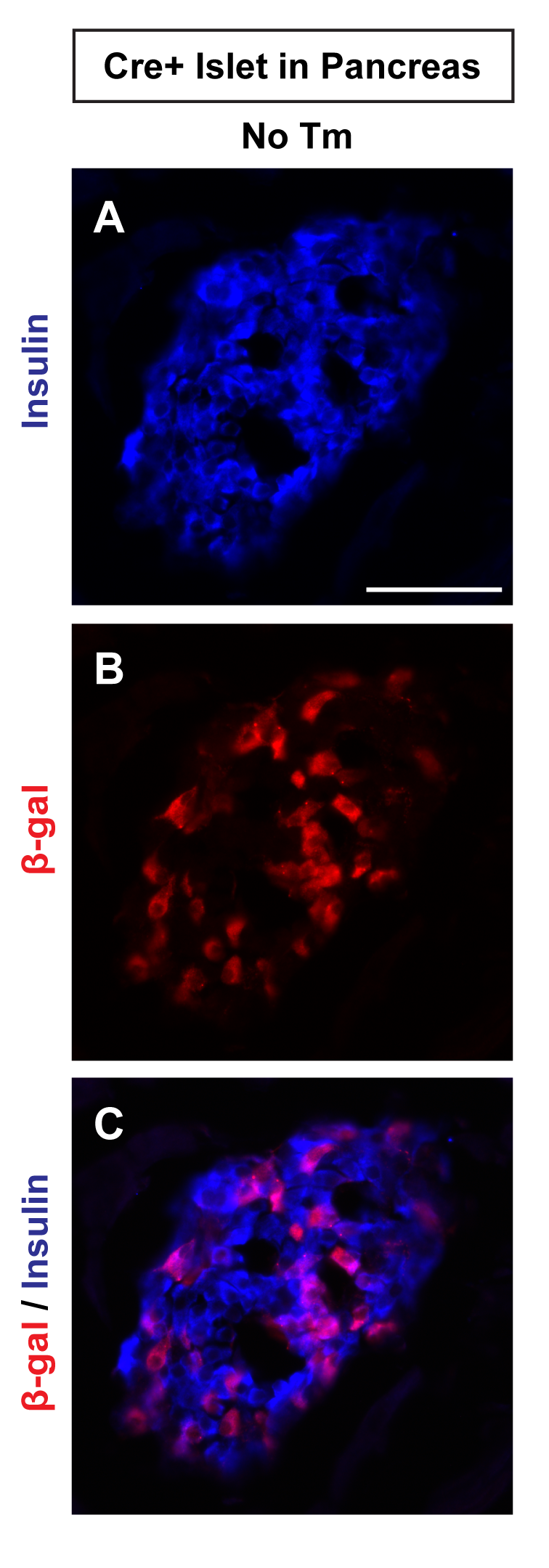

Supplement: Figure S1 — Tamoxifen-independent recombination in RIP - CreERTm ; R26RlacZ mice. Pancreas was harvested from adult RIP-CreERTm;R26RlacZ mice not exposed to tamoxifen (Tm). Immunofluorescence was performed on cryosections for insulin (blue; A, C) and β-galactosidase (β-gal, red; B, C). Scale bar in A is 50 µm, and applies to panels B–C. (TIF) [file pone.0033529.s001.tif]

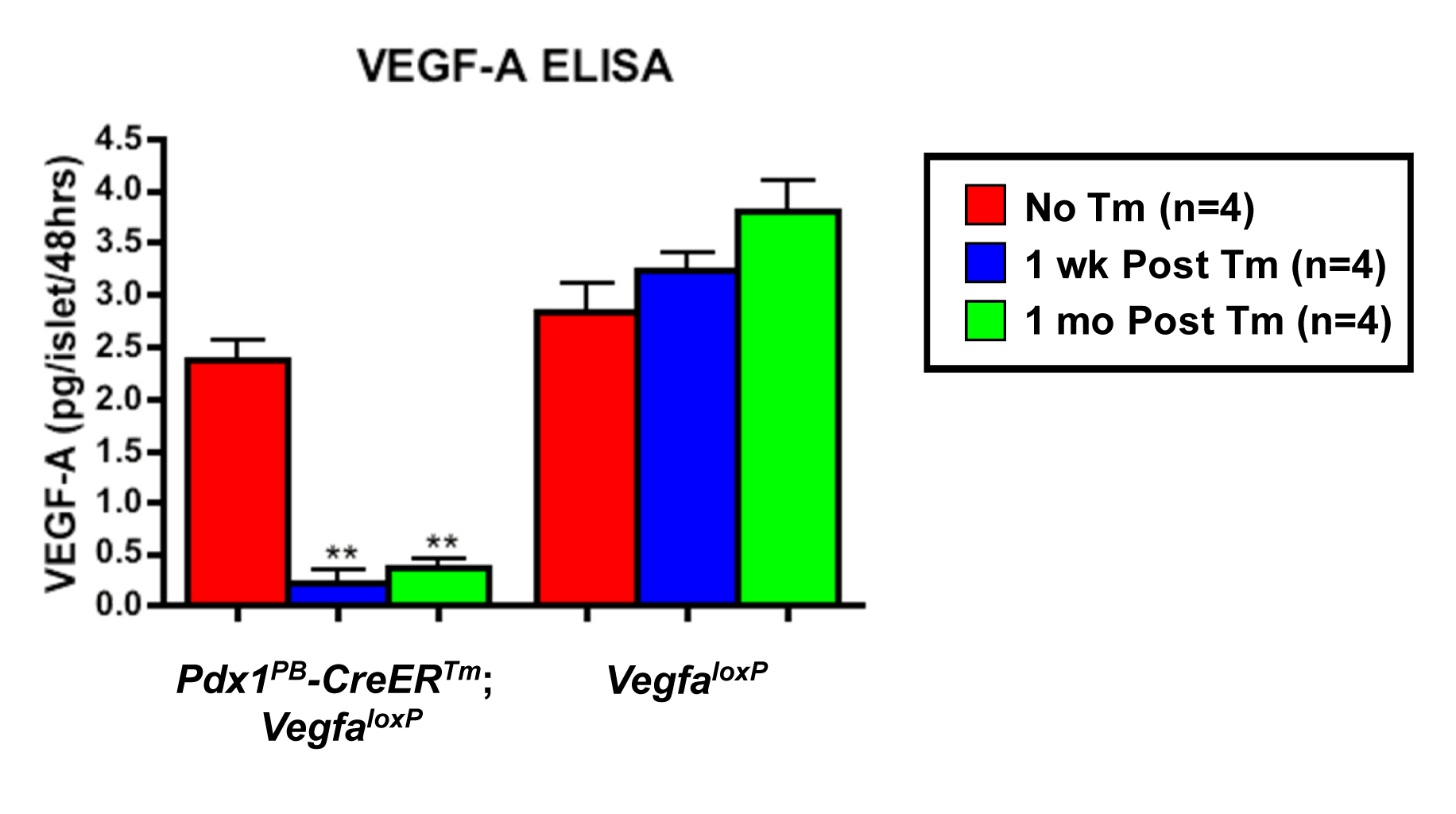

Supplement: Figure S2 — VEGF-A expression is significantly reduced in Pdx1PB - CreERTm ; VegfaloxP islets after administration of 3×8 mg tamoxifen. Islets were isolated from adult Pdx1PB-CreERTm;VegfaloxP mice and VegfaloxP controls before tamoxifen (Tm) treatment and 1 week and 1 month following 3 doses of 8 mg Tm. Aliquots of 70 size-matched islets were cultured in 500 µl RPMI-1640 media for 48 hours at 37°C, and VEGF-A in the cultured media was measured by ELISA (R&D Systems) as described [78]. **P<0.0001. (TIF) [file pone.0033529.s002.tif]

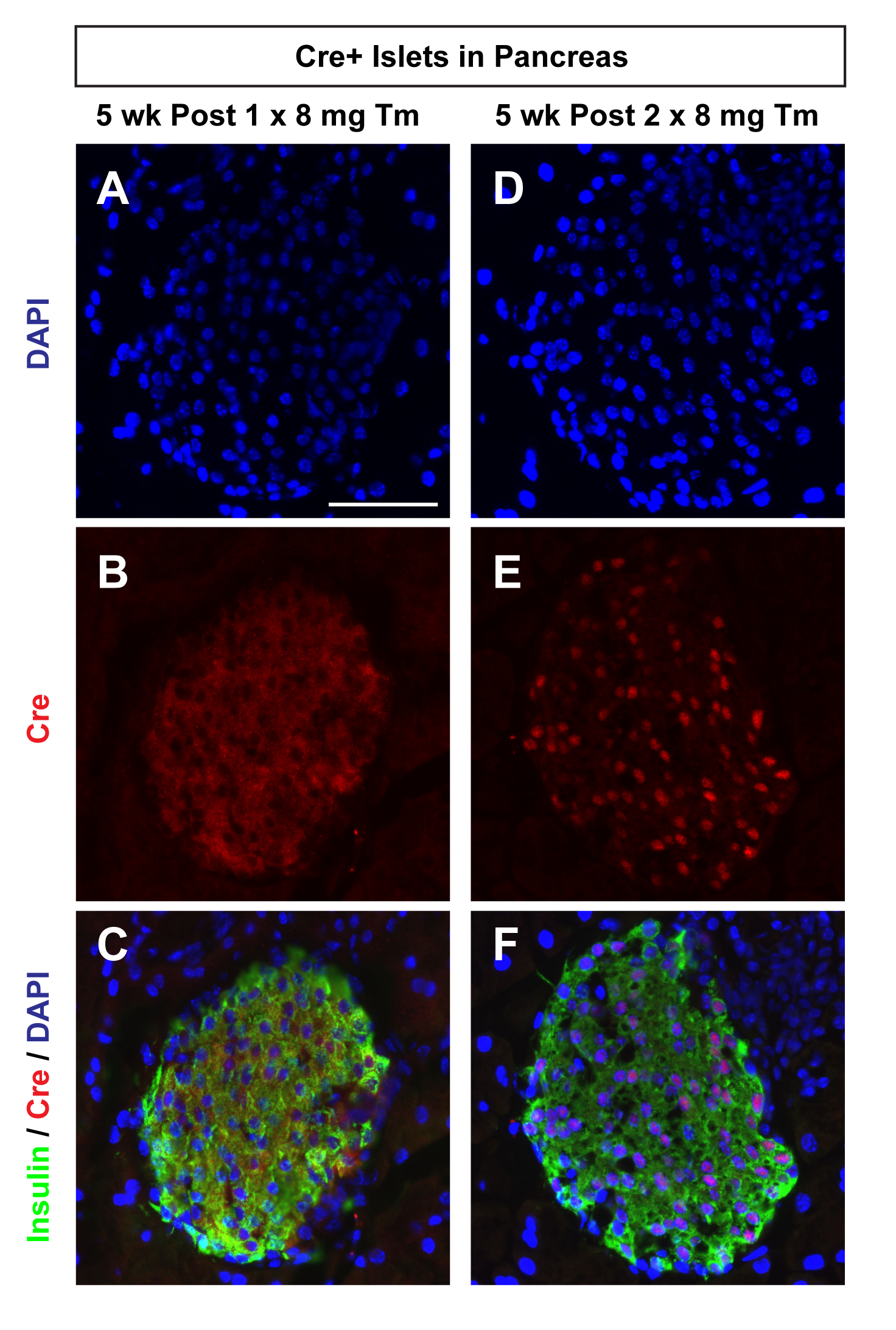

Supplement: Figure S3 — Tamoxifen-induced Cre subcellular localization is dose-dependent. Representative islets from adult Pdx1PB-CreERTm;R26RlacZ mice given one (A–C) or two (D–F) subcutaneous injections of 8 mg tamoxifen (Tm). Pancreata were harvested 5 weeks following the last injection and labeled with antibodies against insulin (green; C, F) and Cre recombinase (red; B, C, E, F); DAPI nuclear stain (blue; A, C, D, F). Scale bar in A is 50 µm, and applies to panels B–F. (TIF) [file pone.0033529.s003.tif]

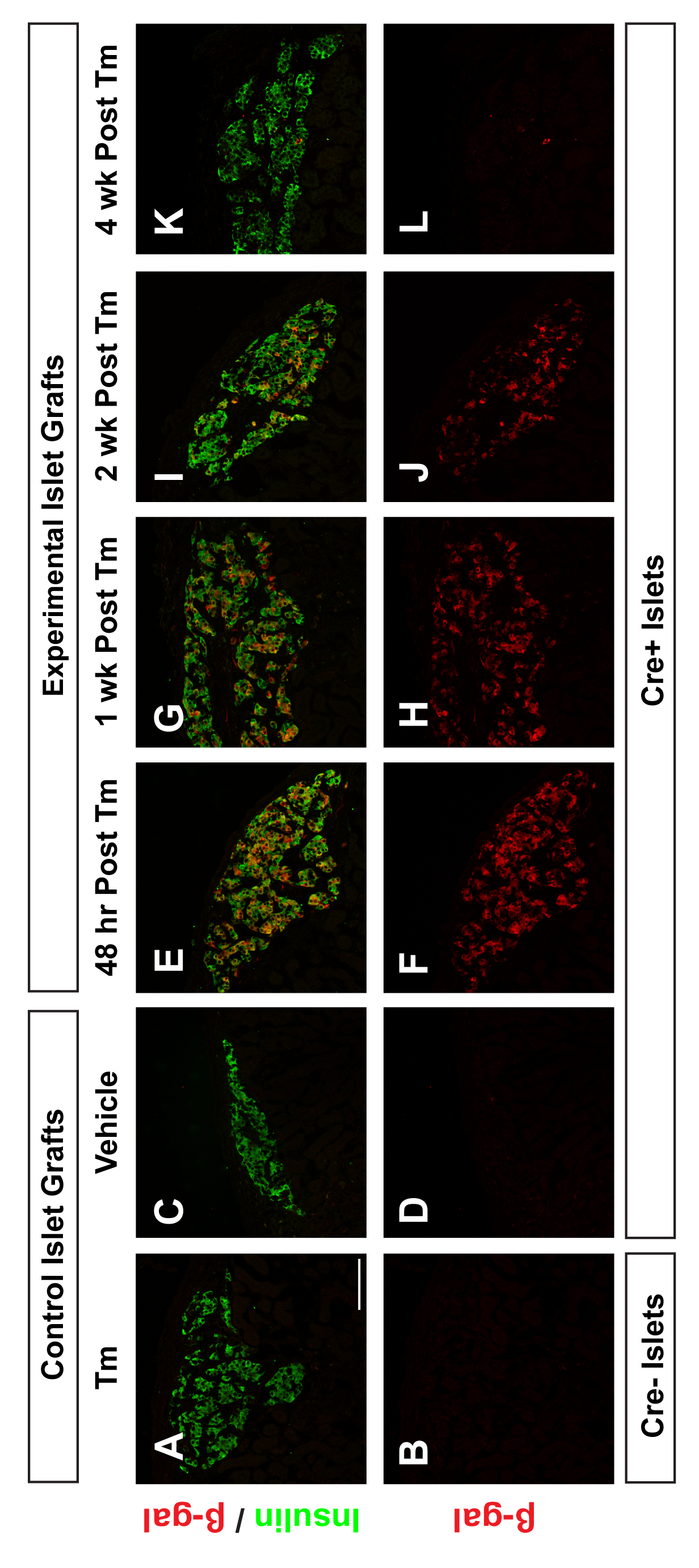

Supplement: Figure S4 — Higher dose tamoxifen induces recombination weeks following administration. Images of the full graft cross-sections shown in Figure 2, before cropping and rotating for visual clarity. Scale bar in A is 200 µm, and applies to panels B–L. (TIF) [file pone.0033529.s004.tif]

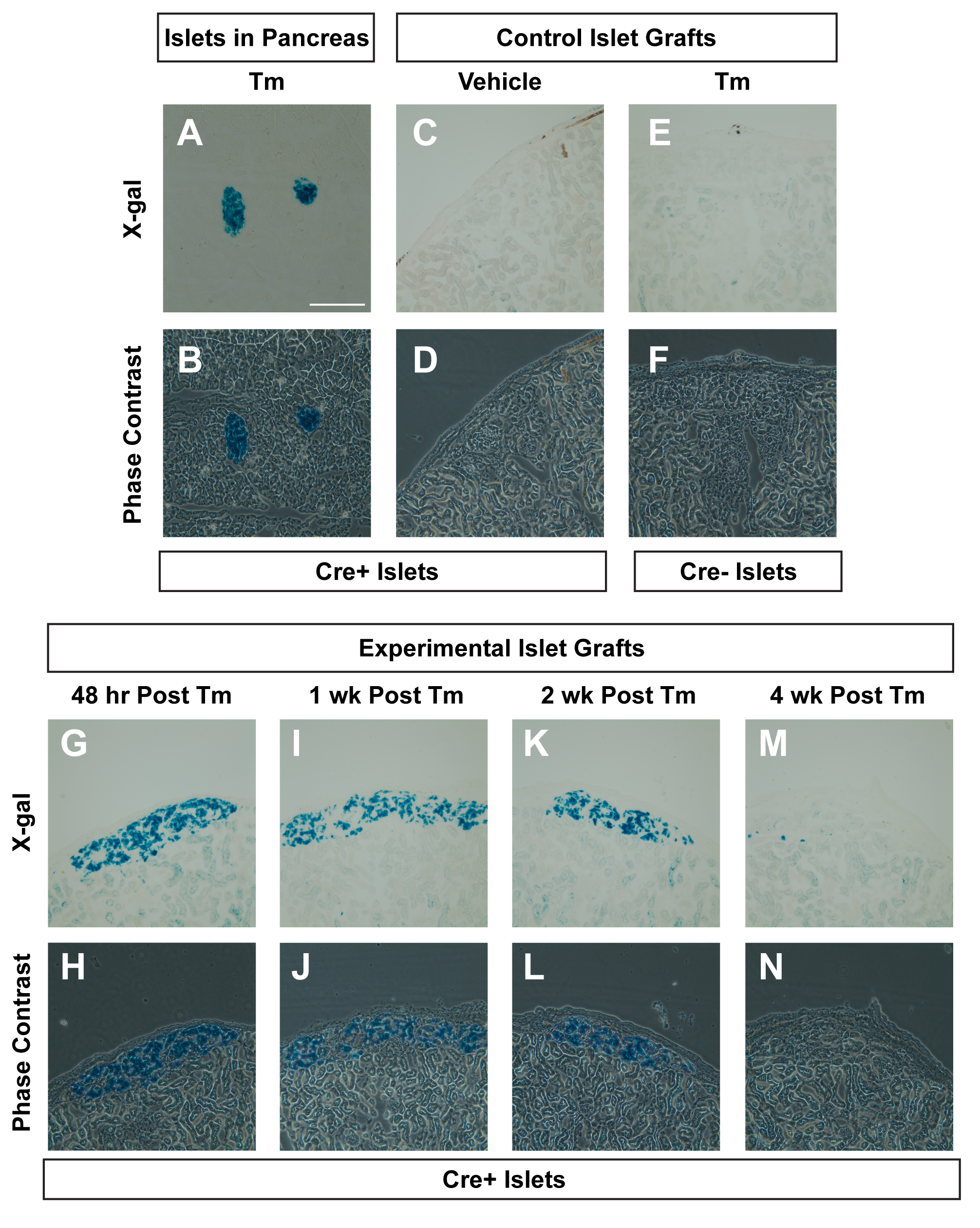

Supplement: Figure S5 — Higher dose tamoxifen induces recombination weeks following administration. Islets from untreated Pdx1PB-CreERTm;R26RlacZ mice (Cre+) or R26RlacZ controls (Cre−) were transplanted into mice given three subcutaneous injections of 8 mg tamoxifen (Tm) or corn oil vehicle at the indicated times following the last injection. Islet grafts were harvested 2 weeks after transplantation and subjected to X-gal staining (A, C, E, G, I, K, M). Phase contrast images with color overlay are shown in B, D, F, H, J, L, and N. Scale bar in A is 200 µm, and applies to panels B–N. Panels C, E, G, I, K, and M are images of the full graft cross-sections shown in Figure 2, before cropping and rotating for visual clarity. (TIF) [file pone.0033529.s005.tif]

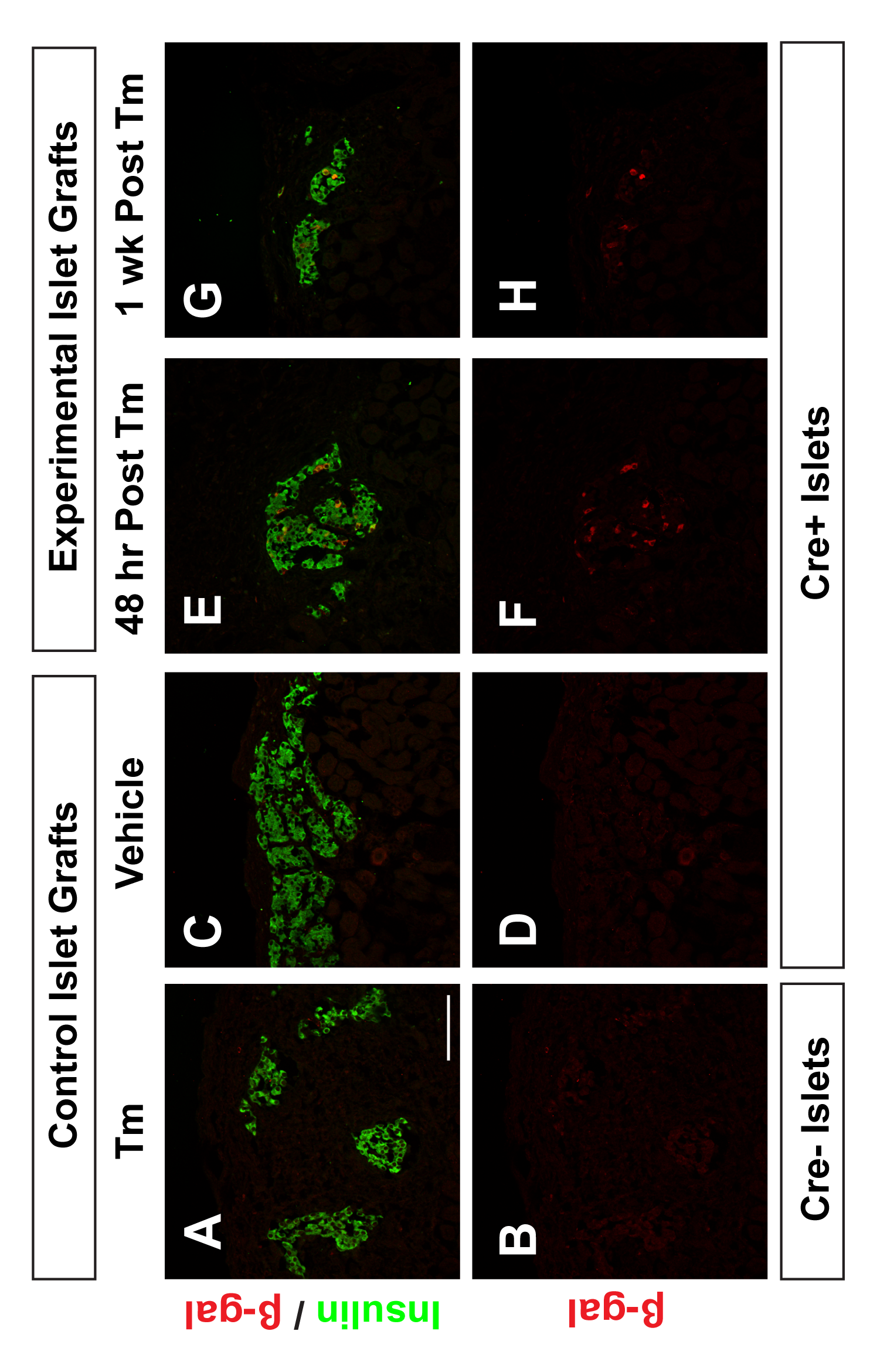

Supplement: Figure S6 — Lower dose tamoxifen induces recombination up to one week following administration. Images of the full graft cross-sections shown in Figure 3, before cropping and rotating for visual clarity. Scale bar in A is 200 µm, and applies to panels B–H. (TIF) [file pone.0033529.s006.tif]

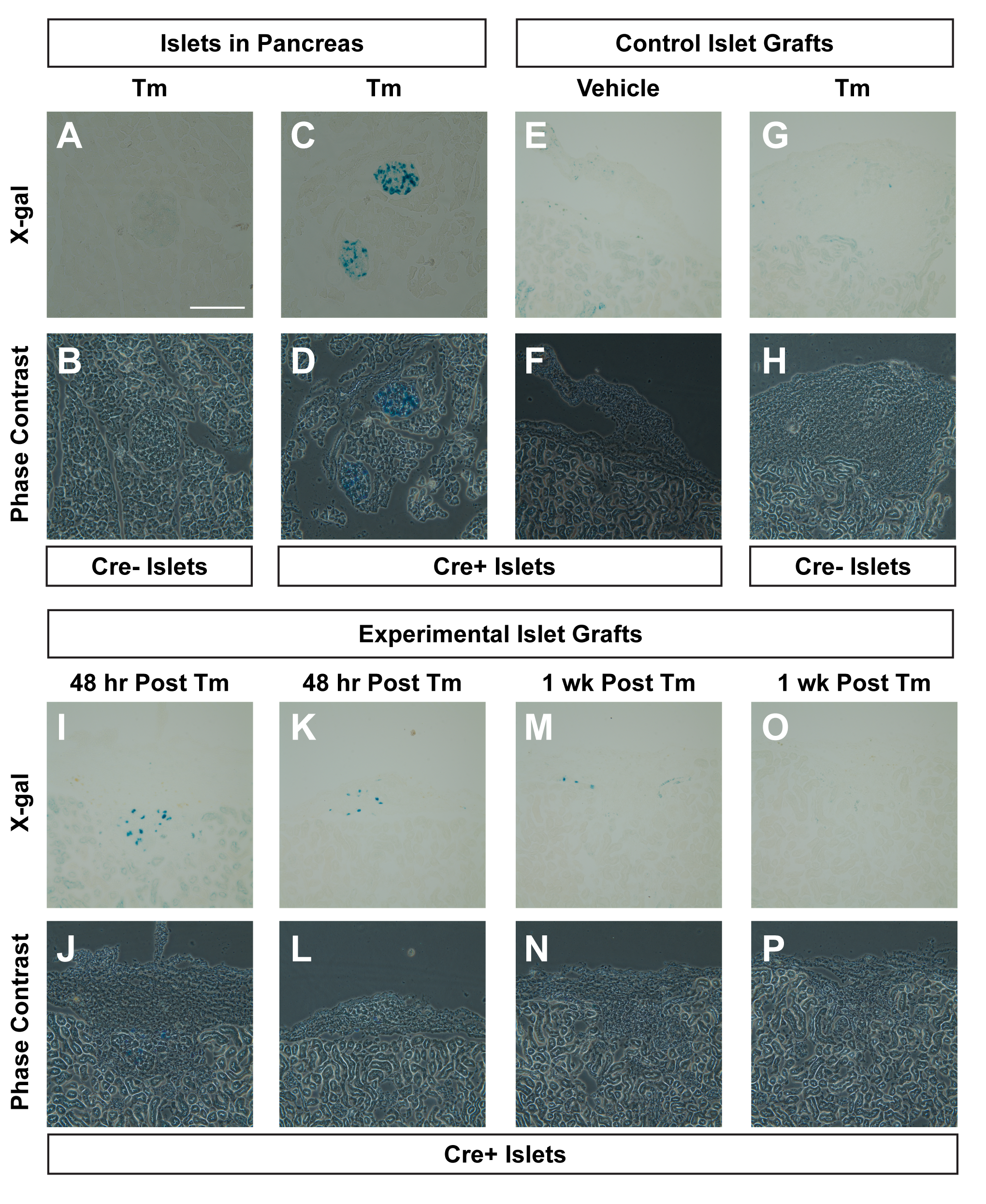

Supplement: Figure S7 — Lower dose tamoxifen induces recombination up to one week following administration. Islets from untreated Pdx1PB-CreERTm;R26RlacZ mice (Cre+) or R26RlacZ controls (Cre−) were transplanted into mice given three subcutaneous injections of 1 mg tamoxifen (Tm) or corn oil vehicle at the indicated times following the last injection. Islet grafts were harvested 2 weeks after transplantation and subjected to X-gal staining (A, C, E, G, I, K, M, O). Phase contrast images with color overlay are shown in B, D, F, H, J, L, N, and P. Scale bar in A is 200 µm, and applies to panels B–P. Panels E, G, I, K, M, and O are images of the full graft cross-sections shown in Figure 3, before cropping and rotating for visual clarity. (TIF) [file pone.0033529.s007.tif]

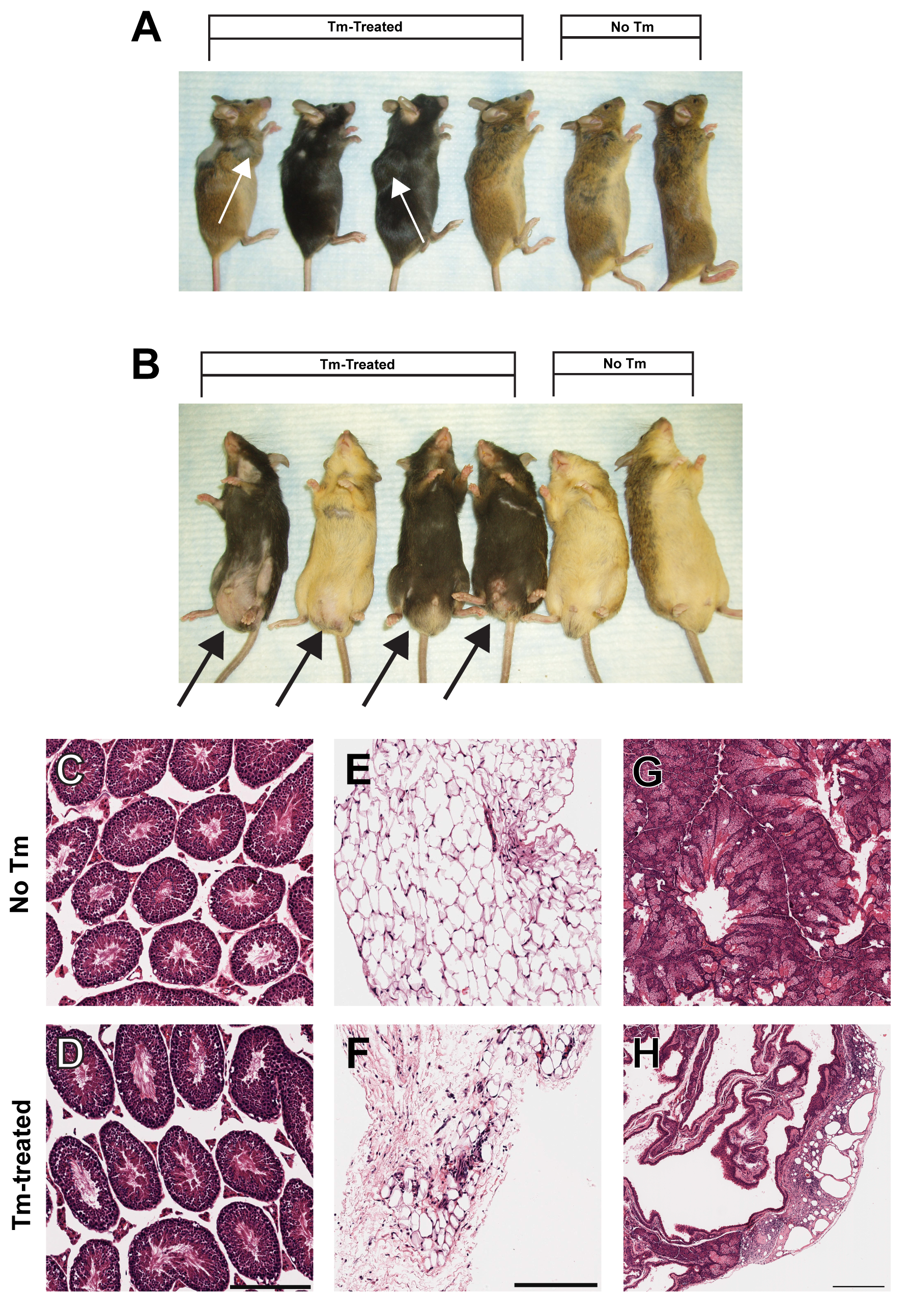

Supplement: Figure S8 — Side effects of tamoxifen treatment. A. Some mice given subcutaneous injections of corn oil vehicle with or without tamoxifen (Tm) had subcutaneous accumulation of oil (white arrows) weeks and months following the last oil injection. B. Tm-treated male mice demonstrated scrotal enlargement (black arrows) as early as one week after treatment and lasting for months. Tm-treated mice shown were given 3×8 mg Tm, but similar results were observed in mice given 3×1 mg Tm. C–H. H&E-stained sections of tissue from the scrota of control (C, E, G) and Tm-treated (D, F, H) male mice. Images were acquired with a ScanScope CS slide scanner. C–D. Seminiferous tubules of the testes. Scale bar in D is 200 µm, and applies to panel C. E–F. Scrotal fat in a control mouse (E), compared to the reactive fat observed in a Tm-treated mouse (F). Scale bar in F is 200 µm, and applies to panel E. G–H. One Tm-treated mouse showed chronic inflammation and fat necrosis within the preputial gland (H); control gland, G. Scale bar in H is 400 µm, and applies to panel G. (TIF) [file pone.0033529.s008.tif]
