# Supplementary material for: Tamoxifen-Induced Cre-loxP Recombination Is Prolonged in Pancreatic Islets of Adult Mice
Source: PLoS One. 2012 Mar 28;7(3):e33529. doi: 10.1371/journal.pone.0033529 (PMC3314663; doi:10.1371/journal.pone.0033529)
Supplement: Table S1 — Summary of mouse strains. (DOC) [file pone.0033529.s009.doc]

| **MGI Nomenclature** | **Abbreviation** | **Reference** |
| --- | --- | --- |
| *Tg(Pdx1-cre/ERT)^1Mga^* | *Pdx1^PB^-CreER^Tm^* | [49] |
| *Tg(Ins2-creEsr1)^1Dam^* | *RIP-CreER^Tm^* | [22] |
| *Gt(ROSA)26Sor^tm1Sor^* | *R26R^lacZ^* | [50] |
| *Vegfa^tm2Gne^* | *Vegfa^loxP^* | [51] |
